# Poly (Vinyl Alcohol) Assisted Synthesis and Anti-Solvent Precipitation of Gold Nanoparticles

**DOI:** 10.3390/nano10122359

**Published:** 2020-11-27

**Authors:** Zhen Liu, Olivia L. Lanier, Anuj Chauhan

**Affiliations:** Department of Chemical and Biological Engineering, Colorado School of Mines, Golden, CO 80401, USA; zhenliu@mymail.mines.edu (Z.L.); olivialanier@mines.edu (O.L.L.)

**Keywords:** gold nanoparticle synthesis, Turkevich method, poly (vinyl alcohol), anti-solvent precipitation

## Abstract

Gold nanoparticles (GNPs) are commonly synthesized using the Turkevich method, but there are limitations on the maximum concentration of gold nanoparticles that can be achieved using this method (often < 1 mM (=0.34 mg/mL) gold precursor loading). Here, we report an inverse Turkevich method which significantly increases the concentration of gold nanoparticles (up to 5-fold) in the aqueous phase by introducing poly (vinyl alcohol) (PVA) to the synthesis system for stabilization. The aim of this study is to understand the effect of PVA and other synthesis parameters, such as trisodium citrate and tetrachloroauric acid concentration, with the goal of maximizing concentration while maintaining gold nanoparticle morphology, stability, and narrow size distribution. The size distribution of GNPs is investigated for a range of parameters by dynamic light scattering and electron microscopy, and ultraviolet-visible (UV–vis) spectroscopy is also utilized to explore the localized surface plasmon resonance (LSPR). Further, the interaction between GNPs and PVA is investigated by Fourier-transform infrared spectroscopy. In addition to increasing the gold loading by varying synthesis parameters, we also develop a novel anti-solvent precipitation method for the PVA-coated GNPs, which enables continuous condensation and purification of GNPs by forming a gold/PVA nanocomposite.

## 1. Introduction

Gold nanoparticles (GNPs) have been extensively investigated due to numerous potential applications in subwavelength optical devices [1,2,3,4], nonlinear optics [5,6,7], optical data storage [8,9], photovoltaic systems [10,11,12,13], surface-enhanced spectroscopy and catalysis [14,15], biological labelling and sensing [16,17], and even cancer therapy [18]. The most common method for synthesis of GNPs, the Turkevich method, is based on the reduction of tetrachloroauric acid (HAuCl_4_) with trisodium citrate to produce GNPs [19,20,21]. Owing to the considerable interest in Turkevich method, a number of researchers have focused on understanding the mechanisms and optimizing the Turkevich method for controlling the particle size and distribution [22,23,24,25]. In the Turkevich method, citrate acts as the reducing agent and as a protector against aggregation by adsorbing on the surface, and additionally plays a third role of controlling the pH since it is a basic salt, which impacts GNP formation [22]. Ojea-Jiménez et al. [26] demonstrated that the sequence of reagent addition is important in obtaining highly monodisperse GNPs, and that using the inverse method will lead to smaller particle sizes. Another study by Zabetakis et al. [27] explored the effect of the concentration of gold ions and trisodium citrate on GNP size and polydispersity, and found that when the gold loading is less than 0.8 mM and Ct/Au > 4, GNPs are monodisperse (PDI < 0.1). Further, Florian Schulz et al. [28] showed that addition of ethylenediaminetetraacetic acid (EDTA) can considerably improve the Turkevich method by reducing the coefficient of variation; however, the mechanism of EDTA addition is unknown and will be investigated in the future. In order to largely increase the scale and yield of GNPs during the synthesis, in 2017, Toit et al. developed a continuous flow synthesis of GNPs under the UV irradiation [29]. In 2020, a rational reactor was designed by Panariello et al., which allowed high reproducibility [30].

Recently, many researchers have explored the synthesis of GNPs in the presence of polymers such as poly (vinyl alcohol) (PVA) in aqueous solutions for optical, biomedical, and catalytic applications. The organic matrices have the desired biocompatibility and mechanical properties for biomedical applications [31,32], and they impart stability to the particles. Porta et al. [33] prepared GNPs for heterogeneous catalysis by reducing tetrachloroauric acid using NaBH_4_ in presence of PVA or poly (vinylpyrrolidone) (PVP). The study observed that the mean size of GNPs dropped when incorporated into PVA, due to the stabilization of GNPs obtained by adsorption of the polymer to the growing particle’s surface. Moreover, PVA-based nanofibers containing GNPs were obtained by Jie Bai et al. [34] using an electrospinning method with GNPs synthesized using the Turkevich method in the presence of PVA. In some other studies, researchers synthesized GNPs and separately incorporated the particles into a PVA matrix to prepare GNP-loaded gels for sensor applications. For example, Omidfar et al. [35] incorporated GNPs in PVA to develop a highly sensitive electrochemical immunosensor, and Raj et al. [36] incorporated GNPs into PVA for mercury detection with a detection range from 1 × 10^−6^ to 2.5 × 10^−5^ M. The presence of the polymer PVA in solution impacts the process of GNP formation in multiple ways. The polymer can adsorb on the GNP surface acting as a protector against aggregation allowing synthesis of smaller nanoparticles [33]. Additionally, the high viscosity of the polymeric solution may reduce the extent of aggregation as well [34].

The impact of the ratio of chloroauric acid to trisodium citrate has been explored in the Turkevich method [27], but to our knowledge this issue was not explored in the presence of PVA. In previous studies involving the synthesis of GNPs, the resulting concentration is <1 mM; thus, with PVA, stable particles may be achieved at higher concentrations with a narrow size distribution, which may be useful for various applications [37]. Here we focus on preparing GNPs in the presence of PVA for a range of tetrachloroauric acid concentrations (5–20 mM) and tetrachloroauric acid to trisodium citrate ratios (1–18). Our main goal is to explore the effect of these parameters on size and determine the maximum concentration of GNPs that can be achieved by optimizing the tetrachloroauric acid and trisodium citrate concentrations while maintaining particle morphology, monodispersity, and stability. We aim to add to previous studies and further improve the inverse Turkevich method to achieve small particle sizes for biomedical applications, while also studying reasons as to why these changes improve the synthesis. Through these studies, we show that in addition to the three known roles of citrate (reducing agent, surfactant, pH control), there is a fourth role of controlling the Debye length that plays a critical role in stabilization of the GNPs by adsorption of the charged ions. It is also shown that addition of PVA results in GNP stabilization with a shorter Debye length compared to GNPs without PVA. To our knowledge, this is the first report of linking synthesis parameters such as citrate and PVA to Debye length and GNP stabilization in solution. Additionally, we determine whether the PVA presence impacts the rate of GNP formation. Finally, we develop an anti-solvent precipitation method, a simple process for the continuous condensation and purification of GNPs by forming a Au/PVA nanocomposite. This novel method can be used to further increase the concentration of GNPs in solution and can only be used on GNPs which have been synthesized with PVA.

## 2. Materials and Methods

### 2.1. Materials and Instruments

Tetrachloroauric acid monohydrate (HAuCl_4_∙H_2_O, 99.9985% assay with gold contents > 49%) and trisodium citrate dihydrate (Na_3_C_6_H_5_O_7_∙2H_2_O, 99.9% assay) were purchased from Electron Microscopy Service (Fort Washington, PA, USA), and were used with no further purification. Poly (vinyl alcohol) (PVA) was purchased from Sigma Aldrich (St. Louis, MO, USA) (≥99%, fully hydrolyzed, viscosity 11.6–15.4 cps). Isopropyl alcohol was purchased from (PHARMCO by Greenfield Global (Palo Alto, CA, USA) (99%, (CH_3_)_2_CHOH, ACS/USP/NF grade). Transmission Electron Microscopy (TEM) grids with Formvar film were purchased from TED PELLA, Inc. (Redding, CA, USA). Localized surface plasmon resonance spectra were characterized by a UV–vis spectrometer from Avantes Enlightening Spectroscopy (Avantes, Louisville, CO, USA). Transmission electron microscopy (TEM) images of high resolution were captured by FEI Talos F200X (Thermo Fisher Scientific, Hillsboro, OR, USA) and scanning electron microscopy (SEM) images were obtained from FEI Helios Nanolab 600I Focused ion beam scanning electron microscopy (FIB/SEM) (Thermo Fisher Scientific, Hillsboro, OR, USA). FT-IR spectra were characterized by Thermo Scientific^TM^ Nicolet^TM^ iN10^TM^ Infrared Microscopy (Thermo Fisher Scientific, Hillsboro, OR, USA). Glassware used during the synthesis process was cleaned by freshly prepared qua regia solution and rinsed with Milli-Q Deion water (resistivity of 18.2 MΩ cm), which was also used for the preparation of all solutions.

### 2.2. Gold Nanoparticle Synthesis

GNPs were synthesized by the inverse Turkevich method, i.e., gold chloride was added to the citrate solution. An aqueous solution of trisodium citrate and PVA was prepared and heated up to the boiling point with continuous magnetic stirring. After 15 min of boiling, tetrachloroauric acid solution in DI water (5 to 20 mM) was quickly added. Upon addition, the color of the solution changed from yellowish to dark grey within 2 s, and then to ruby red or dark purple in approximately 1 min. Reactions were carried out for 25 min, as previous studies [15,16] report a 25 min reaction time for complete conversion to a dispersion of GNPs.

### 2.3. Further Extraction of GNPs: Anti-Solvent Precipitation

After cooling down to room temperature, isopropyl alcohol (IPA) was added to the solution to cause an immediate phase separation into a PVA-rich phase and water-rich phase. The mixture was subsequently centrifuged for 10 min at 4000 rpm to lead to complete phase separation. The GNPs were completely retained in the PVA-rich phase, as evident from a complete absence of any color in the water-rich phase. The GNPs were re-dispersed in water by mixing the PVA-rich phase with water.

### 2.4. Optical Characterization and Electron Microscopy

Absorption spectra of the GNP dispersion were measured to estimate the size and concentration via the localized surface plasmon resonance (LSPR) peak position and intensity. Particle size distribution was measured by dynamic light scattering (DLS) using Malvern Zetasizer NANO ZS (Malvern, UK). The interaction between GNPs and PVA was characterized by measuring the Fourier-transform infrared spectroscopy (FT-IR) spectra with an attenuated total reflectance (ATR) detector. The nanoparticles were also characterized by transmission electron microscopy (TEM) using FEI Talos F200X under a voltage of 200 kV, and the size distribution of more than 500 particles was analyzed using Image J software (ImageJ bundled with 64-bit Java 1.8.0_172, NIH, Bethesda, MD, USA) and fitted by Igor Pro software (Wavemetrics, Portland, OR, USA) [38]. The structure of the GNPs/PVA nanocomposite was characterized by SEM after milling a flat surface using Ga ions and depositing a thin Pt layer using FEI Helios Nanolab 600I focused ion beam scanning electron microscopy (FIB/SEM).

## 3. Results and Discussion

### 3.1. PVA Speeds up Formation of Gold Nanoparticles

The color of the gold colloidal suspension depends on the size and shape of GNPs, which will affect the wavelength of light that is scattered and absorbed; thus, evolution of color with time is a qualitative measure of the rate of particle formation. It is well accepted that the synthesis process involves multiple reactions: the first of which is oxidation of citrate when the aqueous solution is heated, followed by the reduction reaction of auric salt (AuCl_3_) with tetrachloroauric acid to produce the aurous salt (AuCl), then disproportionation reaction of aurous salt (AuCl), resulting in the formation of gold atoms, which act as the monomers for the formation of the particles [28]. However, a recent study challenged the mechanism above, suggesting that the mechanism includes two sequential reduction steps (Au^3+^→Au^+^→Au^0^) [39]. After the chemical reactions, formed gold atoms nucleate and form nuclei, which then grow to form particles. The particle growth involves a combination of aggregation of particles and growth due to additional gold atoms depositing on the particles.

**Figure 1:**
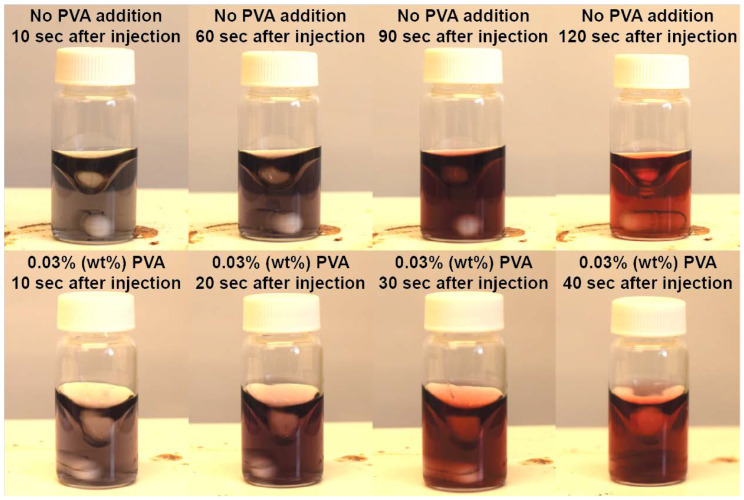
Images of GNP solutions with and without addition of PVA during the synthesis process at different time points after precursor injection (0.6 mM Au precursor, Ct/Au = 6).

As shown in Figure 1, in the absence of PVA, the color of the gold colloidal suspension changed from dark grey to purple and then eventually to ruby red in a total time of approximately 2 min. In the presence of PVA, the color change was much more rapid, with the entire process taking only approximately 40 s. At 40 s, the sample with PVA had a distinct red color, while the one without PVA was considerably darker, indicating the difference between the two cases. The darker color indicates that the GNPs are not fully formed, and the size of the GNP was not fully uniform. Eventually, the color of the gold colloid became ruby red in both cases, with and without PVA, suggesting that the particle sizes in both cases are comparable. While more quantitative comparisons between samples with and without PVA are presented below, this qualitative visual comparison quickly demonstrates that the presence of PVA speeds up the rate of particle formation for reactions with the same concentrations of citrate and gold chloride. The likely reason for the difference in the particle formation rate is the stabilization of small particles by PVA. In this experiment, citrate, which acts as a surfactant and a reducing agent, was in excess. We hypothesize that when PVA was added to the reaction, less citrate was required to stabilize the particles and PVA prevented citrate from adhering to the surface of the particles; thus, more citrate was in the solution to behave as a reducing agent.

### 3.2. Impact of PVA on Particles Size

**Figure 2:**
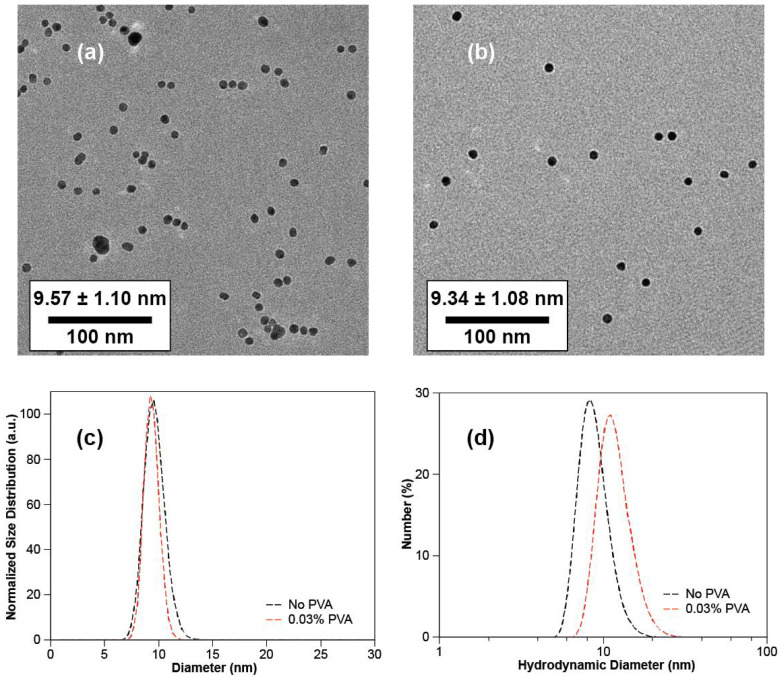
TEM images and size distribution plots of GNPs, 0.6 mM gold ions loading, Ct/Au = 6: (**a**) TEM image of GNPs with no PVA added; (**b**) TEM image of GNPs with 0.03% PVA added during the synthesis; (**c**) physical size distribution of GNPs in Figure 2a,b analyzed from TEM images; (**d**) hydrodynamic diameter distribution of GNPs in Figure 2a,c analyzed from DLS.

The above experiments show that PVA speeds up the rate of particle formation. To explore quantitatively whether PVA affected the final GNP size, particles were again prepared with 0.03% or 0% PVA and identical concentrations of citrate and gold chloride. DLS measurements of particles prepared with 0.6 mM Au and 3.6 mM Ct showed that the number-weighted mean hydrodynamic diameter of the system with PVA was 12.0 ± 2.8 nm compared to the 8.9 ± 1.9 nm for the system without PVA, as shown in Table 1 and Figure 2d. The size distribution of GNPs from DLS is shown in the Supplementary Materials. Size measurements by TEM (Figure 2c) showed that PVA addition results in monodisperse GNPs, and there was only a small change in mean size from 9.57 ± 1.10 to 9.34 ± 1.08 nm (Figure 2a,b). Thus, PVA addition hardly affects the size of GNPs. Note that the hydrodynamic diameter from DLS includes the adsorbed PVA, while the TEM shows the core size of the GNPs. Since TEM imaging showed both sets of GNPs to be of comparable size, the difference of the hydrodynamic diameter measured by DLS must be twice the thickness of the PVA layer, suggesting an adsorbed layer thickness with a mean of 1.6 ± 1.7 nm (estimated thickness). The thicknesses of PVA estimated from volume and intensity-weighted mean hydrodynamic diameters were 2.0 ± 2.3 and 2.8 ± 2.7, respectively (see Table 1).

To further substantiate this data, we compared the zeta potential of the GNPs with and without PVA. The values of the zeta potential of GNPs with and without a PVA layer equaled −24.0 ± 7.5 mV and −44.1 ± 8.0 mV (see Table 1), respectively. Next, we wanted to theoretically calculate the thickness of the PVA layer on the GNP surface using these values for zeta potential. The combination of Poisson Equation [40] and Debye–Hückel approximation [41] was applied to obtain the following expression for the decay of the potential away from the surface:

(1) $$\zeta =\zeta_{0}\exp\left(-\kappa x\right),$$

where ζ (mV) stands for the potential at distance x (nm) away from the surface of the particles, $\zeta_{0}$ (mV) represents the zeta potential of GNPs, and κ (nm^−1^) is the Debye screening wave vector, which equals to the reciprocal of Debye length $\lambda_{D}$ (nm). Debye length $\lambda_{D}$ can be calculated from Equation (2) as 3.74 nm:

(2) $$\kappa^{-1}\left(nm\right)=\sqrt{\frac{\varepsilon k_{B}T}{2\times 10^{3}N_{A}e^{2}I}}{,}_{\;}$$

where $k_{B}$ (m^2^ kg s^−1^ K^−1^) is Boltzmann’s constant, T (K) is temperature, *N_A_* (mol^−1^) is Avogadro’s number, e (H/m) is the elementary charge, and I (mol/L) is the ionic strength of the electrolyte.

To provide an estimate of the thickness of the PVA layer, we assume that the zeta potential of GNPs with PVA (−24.0 mV) is less than that for the GNPs with no PVA (−44.1 mV) only due to the displacement of the slip plane. This assumption may be valid because PVA is neutral and so the potential and charge distribution around the particle may not be significantly impacted by PVA adsorption. Applying this assumption to Equation (1) gives a value of 2.3 nm for the displacement of the slip plane, which can be approximated as the thickness of the PVA layer and is in good agreement with the values obtained by DLS (Table 1). The calculated mean value of PVA thickness from Debye–Hückel approximation is only an estimate because the surface potential could actually be impacted by PVA as well and, additionally, the permittivity of the solution ε is altered by PVA. However, the reasonable agreement between values estimated by this approach and that by DLS and SEM suggests that the assumption is valid.

### 3.3. Effect of Citrate Concentration on Particle Size

Previously, Frens [21] showed that for the original Turkevich method, the ratio of citrate to gold chloride impacts nucleation rates and hence the particle size, allowing the possibility of obtaining GNPs of the desired size ranging from approximately 16 to 150 nm. Previous research also suggests that the dicarboxy acetone intermediate, which is produced during the oxidation of citrate, plays an important role on the morphology and size distribution of final GNPs [24]. Mechanistically, adsorption of the dicarboxy acetone intermediate on the surface of the GNPs stabilizes the GNPs against aggregation due to electrostatic repulsion. Thus, we aimed to study the effect of the citrate to gold chloride ratio in the presence of PVA for the inverse Turkevich method on the formation of the GNPs.

**Figure 3:**
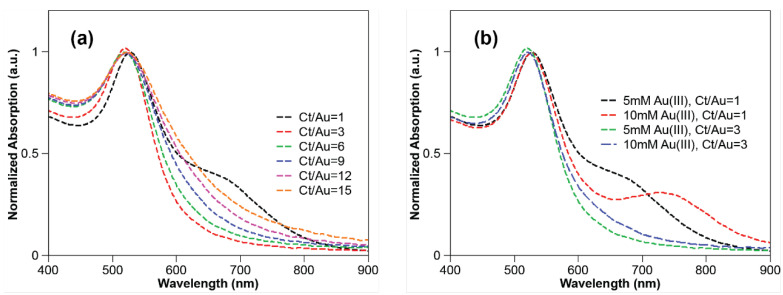
UV–vis spectra of gold colloidal suspensions: (**a**) 5 mM gold ions, Ct/Au varying from 1 to 15; (**b**) with variable of gold loading under the controls of Ct/Au = 1 and of Ct/Au = 3.

**Figure 4:**
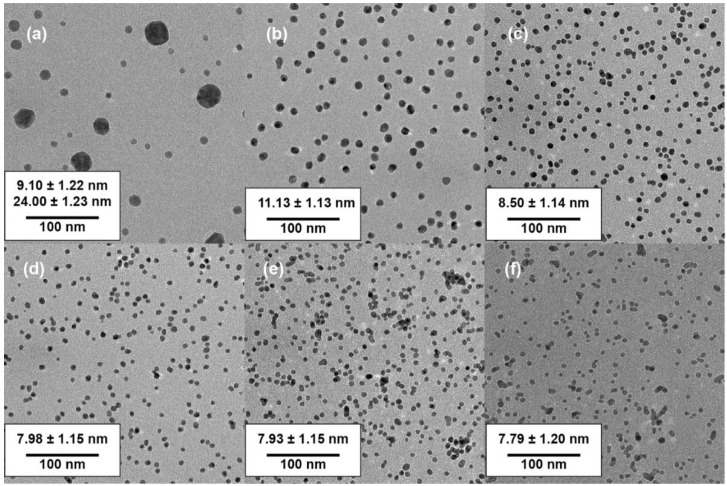
TEM images of GNPs (5 mM gold ions, 0.5% PVA) with variable of Ct/Au and size distribution analyzed using Image J: (**a**) TEM image of GNPs with Ct/Au = 1; (**b**) TEM image of GNPs with Ct/Au = 3; (**c**) TEM image of GNPs with Ct/Au = 6; (**d**) TEM image of GNPs with Ct/Au = 9; (**e**) TEM images of GNPs with Ct/Au = 12; (**f**) TEM image of GNPs with Ct/Au = 15.

**Figure 5:**
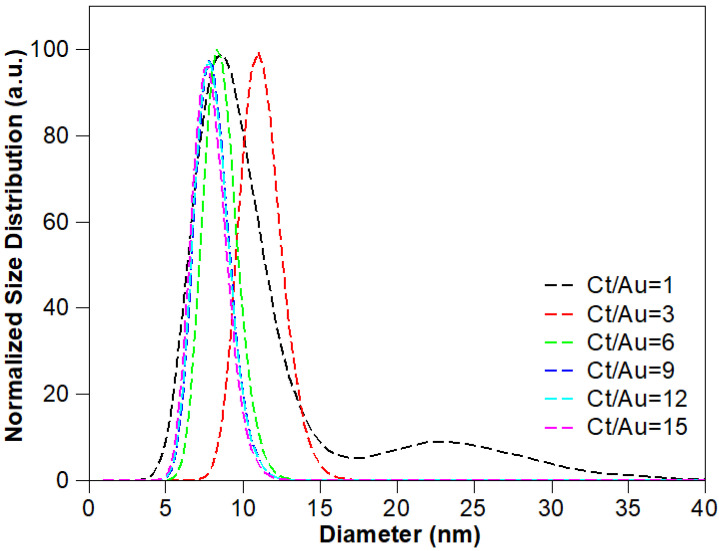
Normalized size distribution of GNPs with increasing Ct/Au (5 mM gold ions, 0.5% PVA).

A series of experiments was conducted for testing the role that trisodium citrate played during the synthesis process. First, the concentration of tetrachloroauric acid and PVA were kept fixed at 5 mM and 0.5% (wt%), respectively, while the concentration of trisodium citrate was varied from 5 to 90 mM (i.e., Ct/Au ratios ranging from 1 to 18). The LSPR absorption (Figure 3) and TEM imaging (Figure 4 and Supplementary Materials) were used to explore the effect of the changing concentrations on the particle formation. And the size distribution histograms of GNPs were analyzed from TEM images, which can be seen from Figure 5. The spectra in Figure 3a show that the LSPR peak position blue shifted when Ct/Au increased from 1 to 6, while the LSPR peak position red shifted when Ct/Au continuously increased from 6 to 15. It was also shown that the plasmon band was the narrowest when Ct/Au equals 3, from which we can estimate that GNPs are of the lowest polydispersity due to the natural properties of LSPR. When Ct/Au equaled 1, there were two plasmon bands at approximately 525 and 720 nm, which implied that GNPs of two populations with different mean sizes were obtained. When Ct/Au was varied from 3 to 15, the absorption of the near-infrared spectrum increased, which indicated aggregation of GNPs. These observations suggest that increasing citrate concentration has the expected and desired effect of decreasing particle size by preventing aggregation, but the trend is reversed at higher concentrations. At higher concentrations, more salt is introduced, which leads to the observed aggregation.

**Figure 6:**
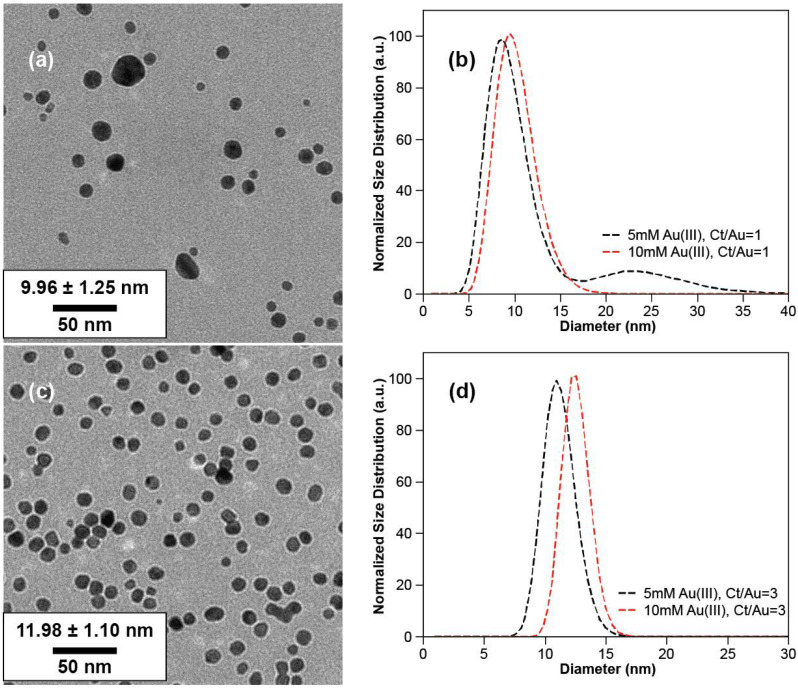
TEM images of GNPs and size distribution histograms: (**a**) 10 mM gold ions, Ct/Au = 1; (**b**) size distribution of GNPs, Ct/Au = 1; (**c**) 10 mM gold ions, Ct/Au = 3; (**d**) size distribution, Ct/Au = 3.

Next, experiments were conducted while keeping the Ct/Au ratio fixed at a constant value and while varying both concentrations and with 0.5% PVA. As shown in Figure 3b, when Ct/Au was fixed at 1 and gold and citrate loading was varied from 5 to 10 mM, the spectra became bimodal suggesting that at the 10 mM concentration, GNPs of two different sizes are formed. This bimodal distribution was further confirmed by TEM images shown in Figure 6. The data in Figure 3b also shows that, when Ct/Au was controlled as 3, the LSPR spectrum did not become bimodal for citrate concentrations as high as 15 and 30 mM (i.e., gold concentrations of 5 to 10 mM). This suggests that excess citrate is critical to obtaining monodisperse particles.

Transmission electron microscopy (TEM) characterization provided more direct data for the effect of citrate concentration on particle sizes (Figure 4) and size distribution of GNPs can be analyzed as followed (Figure 5). For a citrate concentration of 5 mM (Ct/Au = 1), GNPs of two different populations were observed in TEM imaging, with mean sizes of 9.10 ± 1.22 nm and 24.00 ± 1.23 nm, respectively (Figure 4a). This matches the bimodal spectra shown in Figure 3. A bimodal distribution occurs under these conditions due to the slow reaction caused by low Ct, causing a secondary nucleation to occur. Upon increasing Ct/Au to 3 (Figure 4b), monodisperse GNPs were synthesized with a mean diameter of 11.13 ± 1.13 nm and a narrow size distribution. This also correlates with the trends in the spectra as the bimodal spectra transitions into a sharp spectrum with a single peak. On further increasing Ct/Au from 3 to 15, the mean diameter decreased from 11.13 ± 1.13 nm to 7.79 ± 1.20 nm, but further increase led to aggregation (Figure 4b–f). When Ct/Au reached more than 12 (Figure 4e,f), more clusters were observed from TEM characterization. These results from TEM analysis match the hypothesis and results from LSPR spectra that increasing citrate leads to smaller particles due to stabilization but eventually the screening of electrostatic interactions dominates. From the collective results, we hypothesize that increasing Ct concentration beyond a critical value reduces stability because of the increasing ionic strength of the solution that leads to shielding of the electrostatic interactions. Thus, an initial increase in Ct concentration leads to smaller particle sizes due to the stabilization effect but eventually the adsorption of citrate on ions is not enough to overcome the decreasing repulsive interactions due to the increasing ionic strength.

The TEM results for the experiments in which Ct/Au was kept fixed matched the LSPR spectra above (Figure 3b). For Ct/Au = 1, the plasmon band broadens and two LSPR peaks are evident in the LSPR graph, which also correlates with the size distribution of GNPs from TEM images from Figure 6a,b. More TEM images of GNPs can be found in the Supplementary Materials for gold loading of 15 and 20 mM. The spectra showed only one LSPR peak at approximately 521 nm when Ct/Au = 3 for the two cases of Ct concentrations at 15 and 30 mM. From TEM analysis, GNPs of a mean diameter of 11.13 ± 1.13 nm could be obtained when 5 mM tetrachloroauric acid was added, and GNPs of a mean diameter of 11.98 ± 1.10 nm were synthesized when 10 mM tetrachloroauric acid was added (Figure 6c,d).

### 3.4. Relating Particle Stability to the Debye Length

Debye length is a simple measure to determine the approximate distance at which particles experience electrostatic interactions. Debye lengths of the gold colloidal suspensions with and without PVA were calculated using Equation (2) and are summarized as Table 2 and Table 3, respectively. These tables include the data summarized above and additional data obtained by conducting the same experiments as described above for Figure 3 except at other concentrations of gold. Each row in these tables correspond to experiments conducted with a fixed Au concentration, while each column corresponds to a fixed ratio of Ct/Au. For each experiment, the color of the solution which is a measure of the size or the state of aggregation is indicated (P = purple; R = red; or A = aggregation) along with the calculated Debye length. The color was determined visually, and aggregation was determined visually as well based on settling of particles. For example, the top row of Table 2 (Au = 5 mM) represents the same data as discussed above in Figure 4a for spectra and in Figure 5 for TEM imaging. When Ct/Au = 1, a gold colloidal suspension of dark purple is achieved due to the size distribution from obtaining GNPs of two populations. The Debye length is calculated as 2.3 nm at this time and no aggregated nanoparticles can be observed from the TEM image (Figure 4a). When Ct/Au was varied from 3 to 9 (Figure 4b–d), a gold colloidal suspension of ruby red was obtained, which indicates the formation of GNPs with lower polydispersity. The Debye length range ranged from 1.7 nm with Ct/Au = 3 to 1.1 nm for Ct/Au = 9. Aggregation was observed from TEM images when Ct/Au reached 9. When Ct/Au changed from 12 to 15 (Figure 4e,f), aggregated GNPs occurred and the gold colloidal suspension appeared dark purple; the dark purple color can be explained as the clusters have a wider range of absorption in visible light than the absorption range from the previous gold suspension. The Debye length of the gold colloidal suspension changed from 0.96 nm when Ct/Au = 12 to 0.87 nm when Ct/Au = 15. However, when the Debye length of the gold colloidal suspension reached less than 0.8 nm, GNPs had a strong tendency for aggregating and precipitating. In other words, GNPs would aggregate when the concentration of salt ions was high enough to screen the electrostatic interactions.

These observations remain qualitatively similar for higher Au concentrations of 10, 15 and 20 mM (rows 2–4 in Table 2) but there are some quantitative differences. Table 2 shows a general trend that the synthesis of GNPs with PVA is stabilized above a critical Debye length of <1 nm. When gold loading is increased, the critical Debye length is also increased, so conditions with less gold precursor have a shorter critical Debye length. This is likely due to less GNPs being generated.

Gold colloidal suspensions prepared with no PVA are shown in Table 3. The highest concentration of gold ions which remained stable was 2 mM, with a calculated Debye length of 2.67 nm. When the Debye length of gold colloidal suspension with no PVA was less than 2.37 nm, GNPs not stabilized. Comparing Table 2 and Table 3, a synthesis system with greater Debye length is necessary for gold colloidal suspensions with no addition of PVA. In other words, the presence of PVA could stabilize GNPs in the suspension with relatively small Debye length, which could reach as low as ~0.87 nm. A smaller Debye length results from a suspension with great ionic strength I, which indicates that PVA provides additional steric stabilization to the gold colloidal suspension.

**Figure 7:**
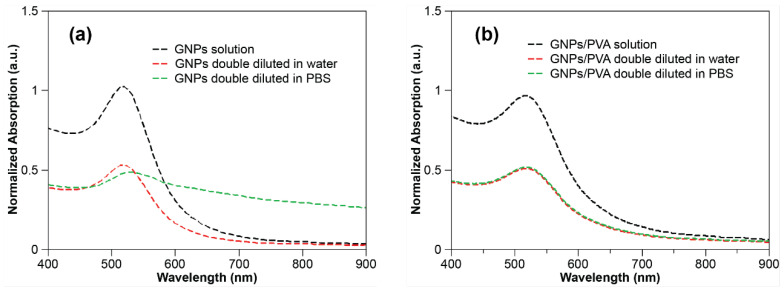
LSPR graphs of gold colloidal suspensions diluted in PBS and DI water: (**a**) 0.6 mM Gold ions, Ct/Au = 6, no PVA addition, double diluted in water and PBS solution, separately; (**b**) 0.6 mM gold ions, Ct/Au = 6, 0.03% PVA addition, double diluted in DI water and PBS solution, separately.

Based on the above results, it can be concluded that the GNPs without PVA aggregate when Debye length becomes smaller than approximately 2 nm, while those with PVA do not aggregate until the Debye length becomes less than approximately 1 nm. To further test this conclusion, we diluted GNP formulations with and without PVA with 1 × PBS. The Debye length of 1× PBS is 0.76 nm, so the Debye length after 1:1 dilution of the GNP sample will be approximately 1 nm, which suggests that the formulation without PVA should be destabilized while the one with PVA should remain stable. Control experiments were conducted by diluting 1:1 with DI water as well, which does not impact the Debye length. UV–vis spectra of both formulations before and after addition of PBS (Figure 7) show that the plasmon band became wider only for PBS dilution of the sample without PVA (Figure 7a) where the sample with PVA remained stable (Figure 7b). This confirms that only the sample without PVA aggregated with the smaller Debye length caused by PBS dilution, and the sample with PVA remained stable. These results show that Debye length plays a role in particle stabilization and that PVA can help to increase particle stabilization through its effect on Debye length. A previous study also showed that Debye length influences the color of the GNP solution [42]. Interestingly, it has been previously shown in the literature that the Debye length plays an important role for behavior of poly (methyl methacrylate) particles and plays a role in determining the density of GNPs deposited on template structures in UV nanoimprint lithography as well [43,44].

### 3.5. Concentrating Particle Concentration via Anti-Solvent Precipitation

**Figure 8:**
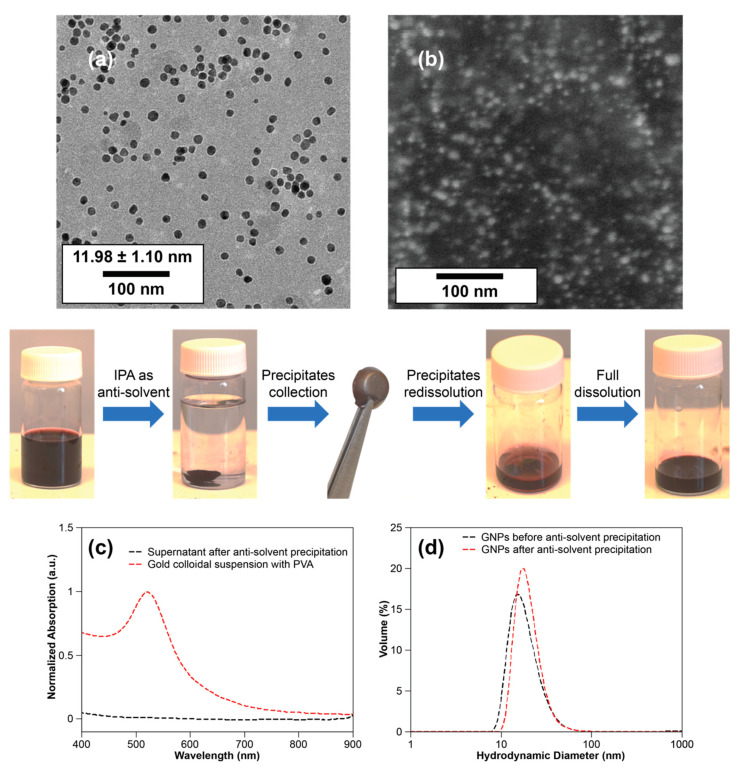
Diagram of anti-solvent precipitation and the re-dissolution process (mid–row): (**a**) TEM image of GNPs (10 mM gold ions, Ct/Au = 3); (**b**) SEM image of Au/PVA nanocomposite obtained from anti-solvent precipitation; (**c**) UV–vis absorption of the gold colloidal suspension with PVA and supernatant after anti-solvent precipitation; (**d**) size distributions of GNPs before and after anti-solvent precipitation from DLS.

Since the maximum precursor used in this study was 20 mM, the maximum concentration of GNPs obtained after the synthesis is 0.4% (wt%). Increasing the concentration of gold in a particle suspension also requires an increase in citrate concentration which decreases the Debye length and results in undesirable effects on the particle morphology. If a higher concentration of gold particles is required for any application, the gold suspension could potentially be concentrated by evaporation of water; however, this method will cause salt concentration to increase as the water evaporates and could lead to GNP aggregation. Increased concentrations of GNPs which are stable will be useful for numerous applications, and in past studies attempts to synthesize highly concentrated metallic particle solutions resulted in large polydisperse particles [45,46]. Alternatively, to address this concern, we propose a method called anti-solvent precipitation that involves precipitation and redissolution of the GNPs/PVA nanocomposite (Figure 8), which we were able to use to increase the concentration up to 4%, which is the equivalent of a synthesis with 200 mM Au loading. 

This is a quick and effective method to extract the GNPs in the solvent which can only be used for GNPs coated with PVA. First, the GNPs/PVA hydrogel was extracted in isopropyl alcohol because of the low solubility of PVA in most organic chemicals or solvents, such as isopropyl alcohol, ethanol, and acetone. The precipitated GNPs/PVA hydrogel was centrifuged and collected; then, further evaporation of the remaining water in the hydrogel was performed by adding heat to the GNPs/PVA nanocomposite. The obtained GNPs/PVA nanocomposite was re-dissolved in small amounts of DI water, which led to increasing the concentration of GNPs up to 10 times compared to the original concentration. TEM images, shown in Figure 8a, were captured for the gold colloidal suspension before the anti-solvent precipitation. A comparison of TEM images, which were captured before and after anti-solvent precipitation, can be found in the Supplementary Materials. SEM images (Figure 8b) were also captured for GNPs/PVA nanocomposite, which showed GNPs were stable and segregated in the polymer matrix. Compared with the gold colloidal suspension, the optical absorption of the supernatant after the anti-solvent precipitation vanished, which indicated that all GNPs were precipitated (Figure 8c). The hydrodynamic diameter measured from DLS remained identical after the anti-solvent precipitation, indicating that shape and the size of the GNPs were maintained, and the GNPs were uniformly suspended in the new solvent without aggregation. Further purification of GNPs can be done by using a cyclic process of anti-solvent precipitation and re-dissolution.

**Figure 9:**
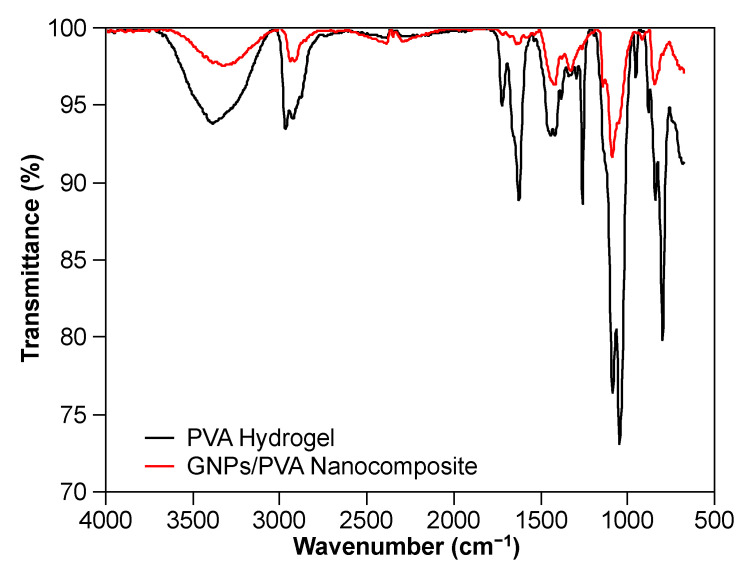
FT-IR spectrum for PVA hydrogel and GNPs/PVA nanocomposite from anti-solvent precipitation.

The interaction between GNPs and PVA is still unknown. However, a numerical MD simulation identified the nonbonded interaction between silver nanoparticles and PVA [47]. Similar to silver nanoparticles, we hypothesize that the nonbonded interaction exists between GNPs and PVA. To investigate this, the interaction was characterized by FT-IR (Figure 9). Both PVA hydrogel and GNPs/PVA nanocomposite were obtained from anti-solvent precipitation by isopropyl alcohol, and their FT-IR spectra were characterized with ATR detector using air as background. It can be clearly observed that the peaks from the PVA hydrogel and the GNPs/PVA nanocomposite are identical, which means no new peak for the GNPs/PVA nanocomposite shows up from the spectrum. Thus, the interaction between GNPs and PVA is nonbonded.

## 4. Conclusions

Here, we report a straightforward method for increasing the concentration of GNPs using the inverse modified Turkevich method by introducing PVA to the synthesis. PVA serves as a stabilizer of GNPs and blocks positive salt ions from attaching on the negatively charged surface of GNPs, which makes it possible to largely increase the concentration of GNPs during the synthesis process. However, if the concentration of the salt is high enough, salt ions can also be transported through the PVA layer and attach on the surface of GNPs, which leads to the aggregation and precipitation of GNPs. The ratio of the concentration of citrate to gold ions was also shown to play an important role by affecting the morphology and the Debye length of GNPs during the synthesis process, and the most monodisperse GNPs were obtained when Ct/Au ≥ 3. LSPR was also characterized by UV–vis spectrometry and the result from LSPR correlates with the size distribution of GNPs obtained from TEM characterization. Plus, it was shown that GNPs with PVA added were stable in solution with a shorter Debye length than GNPs without PVA. Further, anti-solvent precipitation, which involves an extraction and redissolution process, was applied for increasing the concentration of GNPs coated with PVA in solution, and the size of GNPs was shown to be maintained before and after this purification process by TEM characterization. This work will be useful for applications which require high concentrations of GNPs with improved stability, purity and monodispersity such as sensing, catalysis, drug delivery, and more.

## Figures and Tables

**Table 1:** Hydrodynamic diameter (number, volume and intensity weighted) and zeta potential measurements of GNPs from DLS (0.6 mM, Ct/Au = 6) and the estimated thickness of the PVA layer.

Samples	Number (nm)	Volume (nm)	Intensity (nm)	Zeta Potential (mV)
**GNPs, no PVA**	8.9 ± 1.9	10.1 ± 2.5	11.7 ± 2.781.0 ± 22.1	−44.1 ± 8.0
**GNPs, 0.03% PVA added during synthesis**	12.0 ± 2.9	14.0 ± 3.9	17.2 ± 4.7	−24 ± 7.5
**Estimated thickness of PVA**	1.6 ± 1.7	2.0 ± 2.3	2.8 ± 2.7	-

**Table 2:** Debye lengths of gold colloidal suspensions with addition of 0.5% (wt%) PVA. Ct/Au Ratio is varied in columns going to the right, and the amount of Au (III) (mM) is varied going down the rows. All values for Debye length are in nm.

	Ct/Au	1	3	6	9	12	15	18
Au (III)								
5 mM		(P) 2.3	(R) 1.7	(R) 1.3	(R) 1.1	(P) 0.96	(P) 0.87	(A) 0.80
10 mM		(P) 1.6	(R) 1.2	(A) 0.92	(A) 0.78	(A) 0.68	(A) 0.62	(A) 0.57
15 mM		(P) 1.3	(A) 0.98	(A) 0.75	(A) 0.64	(A) 0.55	(A) 0.50	(A) 0.46
20 mM		(P) 1.1	(A) 0.85	(A) 0.65	(A) 0.55	(A) 0.48	(A) 0.44	(A) 0.40

Purple (P): gold colloidal suspension of purple color; Red (R): gold colloidal suspension of red color; Aggregation (A): GNPs aggregated.

**Table 3:** Debye lengths of gold colloidal suspensions with no PVA. Ct/Au Ratio is varied in columns going to the right, and the amount of Au (III) (mM) is varied going down the rows. All values for Debye length are in nm.

	Ct/Au	3	6	9	12	15	18	21
Au (III)								
0.5 mM		(R) 5.33	(R) 4.10	(R) 3.45	(R) 3.04	(R) 2.75	(R) 2.52	(R) 2.35
1.0 mM		(R) 3.77	(R) 2.90	(R) 2.44	(R) 2.15	(A) 1.94	(A) 1.78	(A) 1.66
1.5 mM		(R) 3.09	(P) 2.37	(P) 1.99	(A) 1.76	(A) 1.59	(A) 1.46	(A) 1.36
2.0 mM		(R) 2.67	(P) 2.05	(A) 1.73	(A) 1.52	(A) 1.37	(A) 1.26	(A) 1.17

Purple (P): gold colloidal suspension of purple color; red (R): gold colloidal suspension of red color; aggregation (A): GNPs aggregated.

## Supplementary Materials

The following are available online at https://www.mdpi.com/2079-4991/10/12/2359/s1. Figure S1: TEM images of gold nanoparticles with average physical diameter and standard deviation, 5 mM Au (III), 0.5% PVA: (a) Ct/Au = 1; (b) Ct/Au = 2; (c) Ct/Au = 3; (d) Ct/Au = 4; (e) Ct/Au = 5; (f) Ct/Au = 6; (g) Ct/Au = 9; (h) Ct/Au = 12; (i) Ct/Au = 15. Figure S2: TEM images of gold nanoparticles with average physical diameter and standard deviation, Ct/Au = 1, 0.5% PVA: (a) 15 mM Au (III); (b) 20 mM Au (III). Figure S3: Size distribution of gold nanoparticles analyzed from TEM images from Figure 1 and Figure 2. Respectively: (a) size distribution of gold nanoparticles (Figure S1), 5 mM Au (III), 0.5% PVA; (b) size distribution of gold nanoparticles (Figure S2), Ct/Au = 1, 0.5% PVA. Figure S4: Size distribution of gold nanoparticles without and with PVA addition measured from DLS, 0.6 mM Au (III), Ct/Au = 6: (a) gold nanoparticles without PVA; (b) gold nanoparticles with PVA. Figure S5: TEM images of gold nanoparticles before and after anti-solvent precipitation: (a) before anti-solvent precipitation; (b) after anti-solvent precipitation.

## Abbreviations

GNPs	gold nanoparticles
PVA	poly (vinyl alcohol)
PVP	poly (vinyl pyrrolidone)
TEM	transmission electron microscopy
SEM	scanning electron microscopy
FIB	focused ion beam
DLS	dynamic light scattering
FT-IR	Fourier-transform infrared spectroscopy
Ct	concentration of citrate ions
Au	concentration of gold precursor
