# Supplementary material for: Poly (Vinyl Alcohol) Assisted Synthesis and Anti-Solvent Precipitation of Gold Nanoparticles
Source: Nanomaterials (Basel). 2020 Nov 27;10(12):2359. doi: 10.3390/nano10122359 (PMC7760612; doi:10.3390/nano10122359)
Supplement: Supplementary file 1 [file nanomaterials-10-02359-s001.pdf]

nanomaterials

Supplement Material for:

Poly (Vinyl Alcohol) Assisted Synthesis and Anti-Solvent Precipitation of Gold Nanoparticles

Zhen Liu [1], Olivia L. Lanier [1] and Anuj Chauhan [1,*]

[1] Department of Chemical and Biological Engineering, Colorado School of Mines, Golden, CO 80401, USA.zhenliu@mymail.mines.edu (Z.L.); olivialanier@mines.edu (O.L.)

* Correspondence: chauhan@mines.edu

Received: date; Accepted: date; Published: 27 November 2020

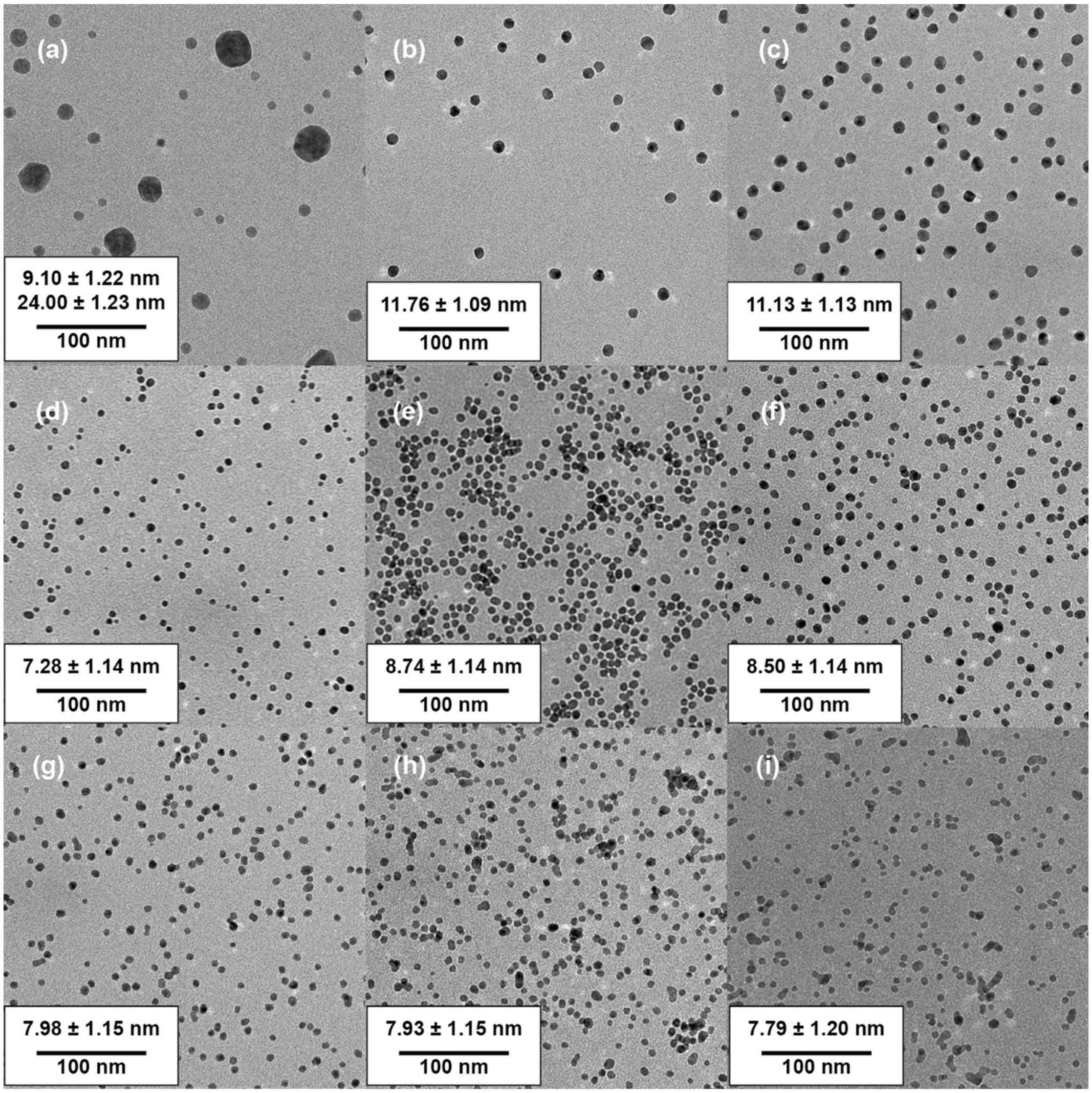

Figure S1. TEM images of gold nanoparticles with average physical diameter and standard deviation, 5 mM Au(III), 0.5% PVA: **(a)** Ct/Au = 1; **(b)** Ct/Au = 2; **(c)** Ct/Au = 3; **(d)** Ct/Au = 4; **(e)** Ct/Au = 5; **(f)** Ct/Au = 6; **(g)** Ct/Au = 9; **(h)** Ct/Au = 12; **(i)** Ct/Au = 15.

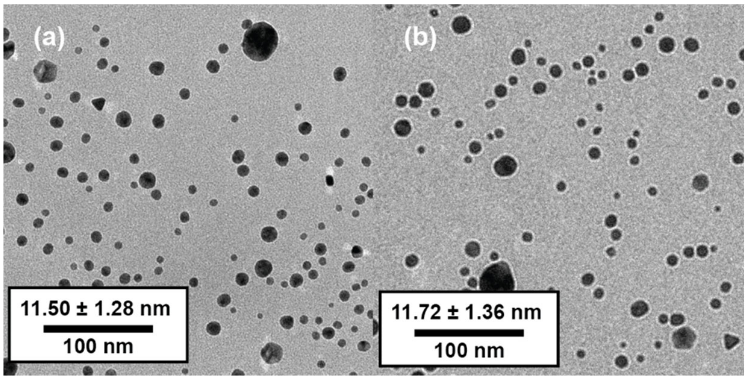

Figure S2. TEM images of gold nanoparticles with average physical diameter and standard deviation, Ct/Au = 1, 0.5% PVA: **(a)** 15 mMAu (III); **(b)** 20 mM Au(III).

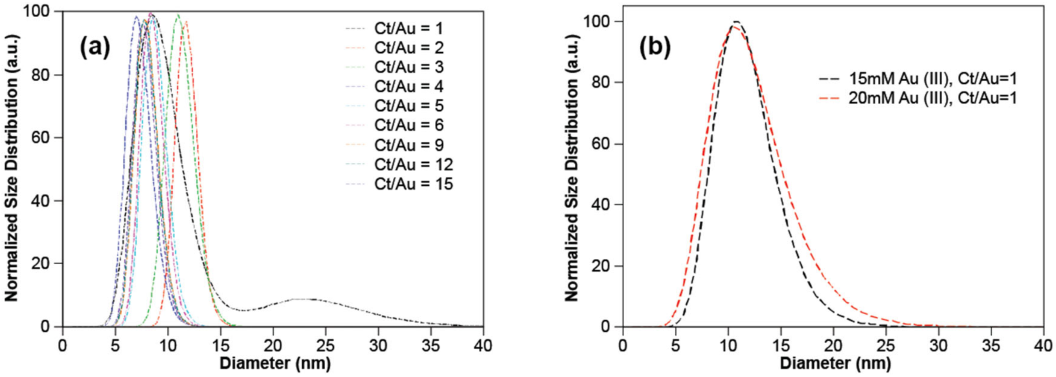

Figure S3. Size distribution of gold nanoparticles analyzed from TEM images from Figure S1 and Figure S2. Respectively: **(a)** size distribution of gold nanoparticles (Figure S1), 5 mM Au(III), 0.5% PVA; **(b)** size distribution of gold nanoparticles (Figure S2), Ct/Au=1, 0.5% PVA.

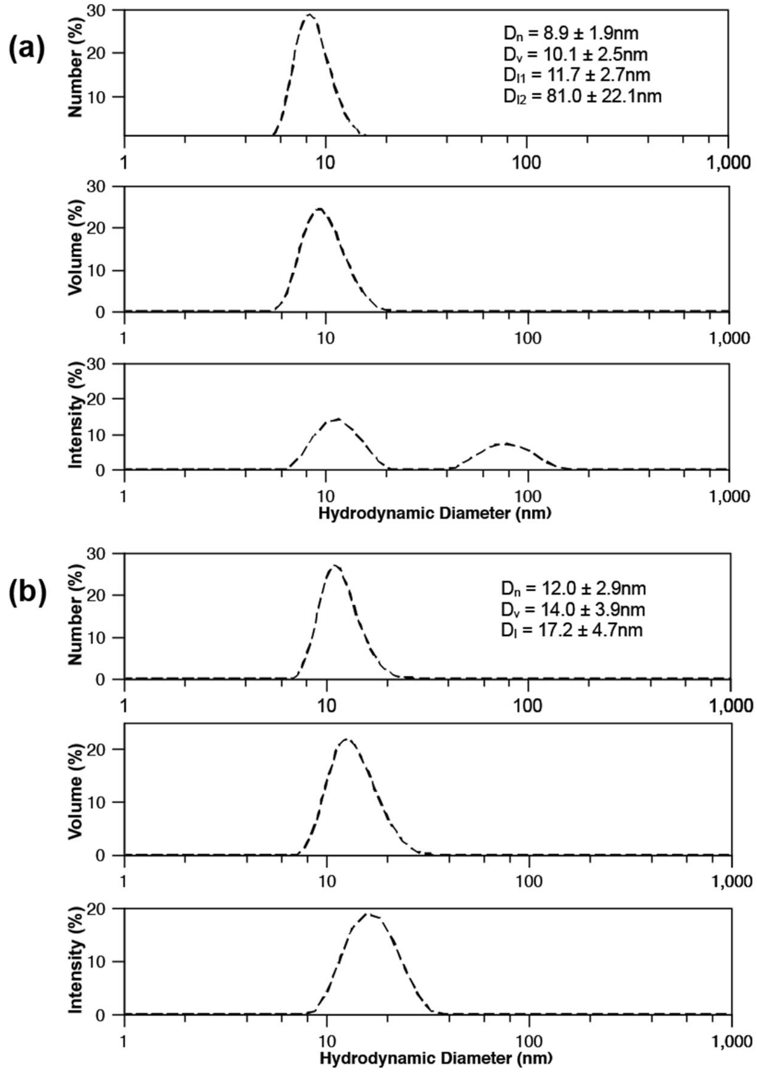

Figure S4. Size distribution of gold nanoparticles without and with PVA addition measured from DLS, 0.6 mM Au(III), Ct/Au = 6: **(a)** gold nanoparticles without PVA; **(b)** gold nanoparticles with PVA.

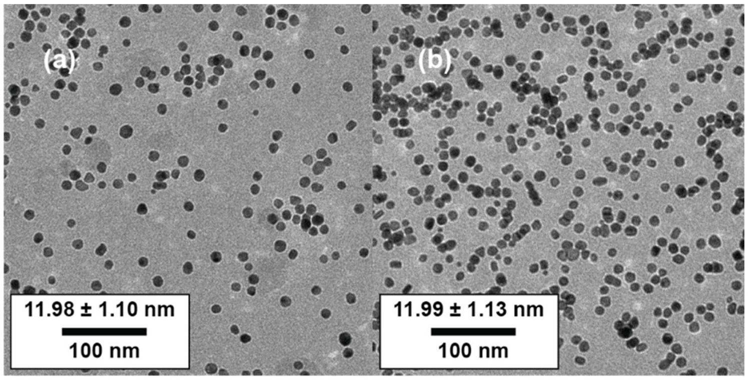

Figure S5. TEM images of gold nanoparticles before and after anti-solvent precipitation: **(a)** before anti-solvent precipitation; **(b)** after anti-solvent precipitation

Publisher's Note: MDPI stays neutral with regard to jurisdictional claims in published maps and institutional affiliations.

© 2020 by the authors. Licensee MDPI, Basel, Switzerland. This article is an open access article distributed under the terms and conditions of the Creative Commons Attribution (CC BY) license (http://creativecommons.org/licenses/by/4.0/).
